# A new method for accurate in vivo mapping of human brain connections using microstructural and anatomical information

**DOI:** 10.1126/sciadv.aba8245

**Published:** 2020-07-29

**Authors:** Simona Schiavi, Mario Ocampo-Pineda, Muhamed Barakovic, Laurent Petit, Maxime Descoteaux, Jean-Philippe Thiran, Alessandro Daducci

**Affiliations:** 1 Department of Computer Science, University of Verona, Verona, Italy.; 2 Signal Processing Lab (LTS5), École Polytechnique Fédérale de Lausanne, Lausanne, Switzerland.; 3 Groupe d’Imagerie Neurofonctionnelle, Institut des Maladies Neurodégénératives, UMR 5293, CNRS, CEA University of Bordeaux, Bordeaux, France.; 4 Sherbrooke Connectivity Imaging Lab (SCIL), Université de Sherbrooke, Sherbrooke, Québec, Canada.; 5 University Hospital Center (CHUV) and University of Lausanne (UNIL), Lausanne, Switzerland.

## Abstract

Diffusion magnetic resonance imaging is a noninvasive imaging modality that has been extensively used in the literature to study the neuronal architecture of the brain in a wide range of neurological conditions using tractography. However, recent studies highlighted that the anatomical accuracy of the reconstructions is inherently limited and challenged its appropriateness. Several solutions have been proposed to tackle this issue, but none of them proved effective to overcome this fundamental limitation. In this work, we present a novel processing framework to inject into the reconstruction problem basic prior knowledge about brain anatomy and its organization and evaluate its effectiveness using both simulated and real human brain data. Our results indicate that our proposed method dramatically increases the accuracy of the estimated brain networks and, thus, represents a major step forward for the study of connectivity.

## Teaser

A new method substantially improves the accuracy of mapped brain networks using anatomy and microstructure informed tractography.

## INTRODUCTION

Tractography based on diffusion-weighted magnetic resonance imaging (DW-MRI) offers the unique opportunity to reconstruct in vivo the major pathways of the brain (*1*) and map the human connectome (*2*). A connectome is typically represented as a graph, where nodes correspond to gray matter cortical areas and/or subcortical nuclei and edges to white matter pathways between them. Because of the gap between the size of the axons (few micrometers) and DW-MRI resolution (few millimeters), each reconstructed pathway, called streamline, does not represent a single axon but rather a group of axons, or bundle, sharing the same path. With this representation, macroscopic brain connectivity can be analyzed using graph theory and network science (*3*, *4*), and this approach has been extensively used to study a wide range of neurological conditions (*5*, *6*).

Despite this potential, a number of technical factors in addition to the complexity of the white matter anatomy introduce ambiguities that are difficult to resolve for tractography, and recent studies raised serious concerns about its effectiveness for studying brain connectivity, as serious biases may be introduced. Thomas *et al.* (*7*) compared tractography reconstructions on high-quality data with axonal tracer results and concluded that their anatomical accuracy is inherently limited, revealing an intrinsic trade-off between sensitivity, i.e., capability of reconstructing real bundles, and specificity, i.e., retrieving only true ones. The international tractography challenge organized by Maier-Hein *et al.* (*8*) highlighted that reconstructions are dominated by false positives and showed that specificity is the main bottleneck. Analyzing topological properties of the networks built with tractography, Zalesky *et al.* (*9*) demonstrated that specificity is actually crucial to study brain connectivity; similar conclusions are drawn in (*10*). Hence, as remarked recently in (*11*), improving the specificity of human connectomes still represents a major challenge in computational neuroscience and may open new avenues toward a more veridical characterization of brain connectivity.

A number of solutions have been recently proposed to improve the accuracy of tractography reconstructions (*12*–*15*). The common idea consists of combining the reconstructed set of streamlines, i.e., tractogram, with signal forward models to assess their actual contribution to the acquired DW-MR images and filter out the most implausible using global optimization techniques. Although the filtered tractograms provide biologically more accurate estimates of connectivity (*16*), none of these methods proved effective in reducing false positives. All solutions are purely data driven and rely only on the acquired DW-MR signal to filter out implausible streamlines. Moreover, streamlines are considered as independent entities, ignoring the fact that in the central nervous system, axons are naturally organized in fascicles. Yet, this fact is explicitly assumed to build a connectome, as streamlines are grouped in bundles and considered as individual edges of the resulting brain network (*2*). However, the definition of “connection strength” for these edges and how to assign them a proper weight are still open questions (*17*). Although some studies that compared tractography with tract-tracing data proved that connectome reconstructions based on the number of streamlines represent a fairly realistic proxy for the connection strength of white matter projections (*18*, *19*), the streamline count should not be confused with the actual fiber count (*17*). Notably, because network models rely on the underlying choices of what an edge represents, the accuracy of tractography reconstructions and how we assign a contribution to the streamlines become crucial.

In this study, we present a novel processing framework for dramatically improving the specificity of the estimated brain networks without affecting their sensitivity. We name it COMMIT2, as it builds on the convex optimization modeling for microstructure informed tractography (COMMIT) (*12*). The original formulation allowed combining tractography with microstructural features of the tissue to enhance the robustness of connectivity estimates, but turned out ineffective for reducing false positives (see Results). We speculate that this information is not enough, and we advocate the need for additional prior knowledge to help tractography in resolving ambiguous configurations and improve the quality of reconstructions.

To this aim, we developed a new formulation that implements two basic observations about the organization of white matter pathways: (i) Streamlines are not “just lines” but represent neuronal fibers, and (ii) these neuronal fibers are naturally organized in bundles. COMMIT2 attempts to recover the connectome that best explains the local axon density estimated from the quantitative DW-MR signal and, at the same time, tries to achieve this goal using the minimum number of bundles. This last condition was inspired by the recent theory about the economy of brain network organization (*20*) and is explicitly promoted in our novel filtering procedure (see Materials and Methods) to reduce the incidence of false positives. Although COMMIT2 shares the same underlying optimization procedure with the original COMMIT, the new formulation is a considerable improvement over the previous one and not only an incremental refinement, as the possibility of injecting anatomical priors represents an important step toward a more veridical estimation of structural connectivity.

## RESULTS

### Sensitivity and specificity of the new formulation on simulated data

**Fig. 1:**
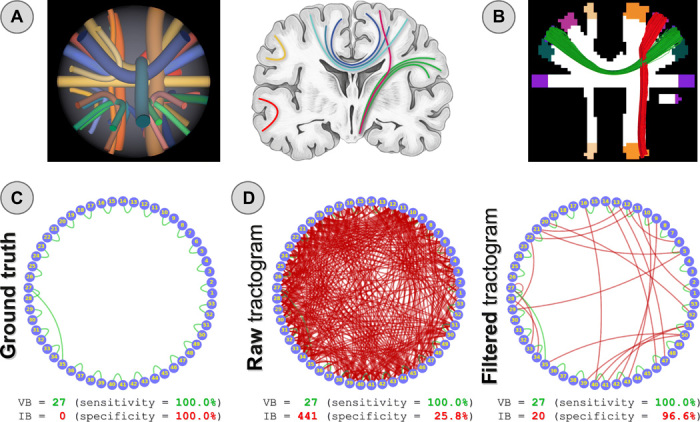
Quantitative evaluation of the proposed method. (**A**) Synthetic dataset (left), inspired by real anatomical bundles of the human brain (right), used to quantitatively evaluate our proposed method. White matter and gray matter masks used for tractography (**B**) and two examples of true-positive (green) and false-positive (red) bundles that can potentially be reconstructed with tractography. Ground truth connectivity represented as a graph (**C**): Blue circles correspond to the 53 gray matter regions shown in (B), whereas green and red arcs represent true-positive (VB) and false-positive bundles (IB), respectively. In (**D**), we compare the sensitivity and specificity of the estimated connectome before (left) and after filtering the tractogram with our COMMIT2 method (right).

**Fig. 2:**
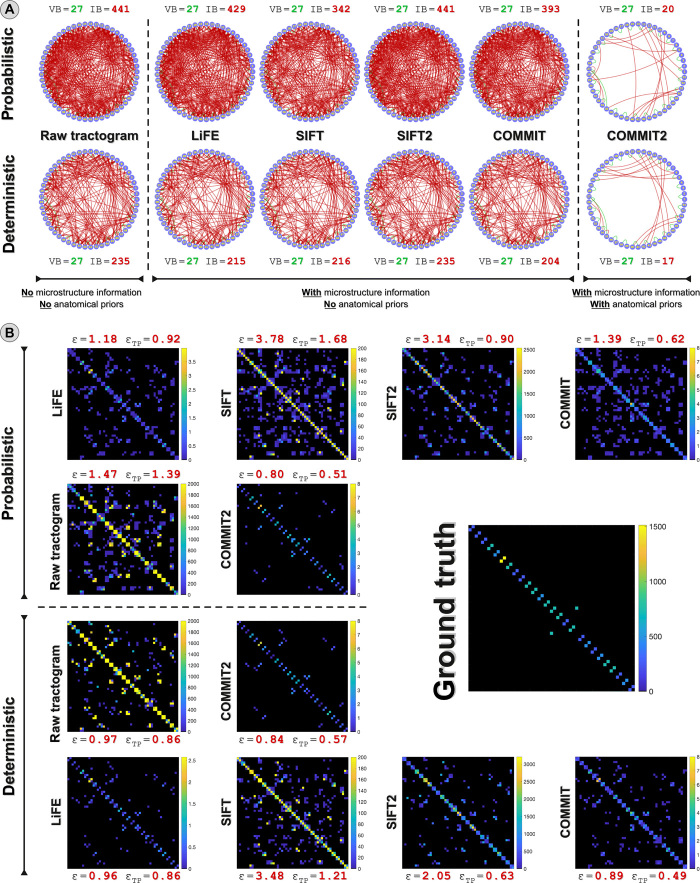
Comparison with state-of-the-art filtering techniques. (**A**) Sensitivity and specificity of the connectomes estimated with tractography before and after applying state-of-the-art filtering methods: LiFE, SIFT, SIFT2, and COMMIT; results with our novel approach are reported in the last column. VBs are reported in green, and IBs are reported in red. (**B**) Ground truth fiber count connectome and weighted connectomes estimated with tractography before and after applying each method. ε and ε_TP_ quantify the difference between the normalized ground truth connectome and those estimated by the methods when considering, respectively, all connections or only the true positives.

To quantitatively assess the effectiveness of our proposal, we used a digital phantom with known ground truth (Fig. 1A) specifically designed to mimic typical fiber configurations encountered in the brain. Figure 1B shows two examples of true-positive and false-positive bundles that may potentially be reconstructed with tractography, while the ground truth connectome is shown in Fig. 1C. We tested both deterministic and probabilistic tractography, and we assessed the sensitivity and specificity of the resulting connectomes using well-established metrics to evaluate tractography (*21*): number of valid bundles (VBs), i.e., true-positive connections, and invalid bundles (IBs), i.e., false positives. Figure 1D reports the number of VB and IB in a representative tractogram reconstructed with probabilistic tractography (left); results hold also for deterministic tractography (Fig. 2A, second row). In line with previous literature (*7*–*9*), all true-positive bundles were recovered, but at the price of including a large number of false positives (IB = 441). After filtering the tractogram with COMMIT2 (right), the IB decreased from 441 to 20, boosting the specificity from 25.8 to 96.6% without affecting the sensitivity.

### Comparison to state-of-the-art filtering techniques

We compared this outstanding performance of COMMIT2 to other techniques that perform similar filtering procedures on the tractograms: linear fascicle evaluation (LiFE) (*13*), spherical-deconvolution informed filtering of tractograms (SIFT) (*14*), SIFT2 (*15*), and COMMIT (*12*). We first tested their effectiveness in removing the false-positive bundles on tractograms reconstructed with both probabilistic and deterministic algorithms, and the results are shown in Fig. 2A. From the first column, we see that both tracking algorithms were able to reconstruct all 27 true bundles, i.e., high sensitivity, but at the price of recovering a large number of false positives, i.e., very low specificity (IB = 441 in case of probabilistic tracking and IB = 235 for deterministic). These results agree with previous literature (*7*–*9*). In columns 2 to 5, we can see neither the sensitivity nor the specificity is substantially affected by filtering methods that use only microstructural information. All tractograms still contain all 27 true bundles, and the number of IB diminished only marginally: LiFE, 441 → 429 and 235 → 215, respectively; SIFT, 441 → 342 and 235 → 216, respectively; and COMMIT, 441 → 393 and 235 → 204, respectively. For this phantom, SIFT2 does not remove bundles, as none of the contributions assigned to the streamlines have exactly zero weight, suggesting that the definition of a threshold is needed to optimally use this method (see further discussion below). On the other hand, the last column clearly shows that the inclusion of anatomical priors, i.e., COMMIT2, has a dramatic impact on the specificity as compared with models that consider only microstructure information: When using COMMIT2, the number of IB is dramatically reduced (441 → 20 and 235 → 17, respectively). These findings were confirmed also using additional digital phantoms with more complex network configurations (fig. S1).

For all the methods, we also compared their ability to accurately estimate the actual edge weights of the ground truth connectome. Figure 2B shows the estimated connectomes represented as matrices, where we can appreciate the quite different definitions of connection strength assumed by each method: actual fiber count used to generate the ground truth phantom, streamline count for the raw and the SIFT-filtered tractograms, and sum of the streamline weights estimated by SIFT2 and LiFE from the entire DW-MR signal or, in the case of COMMIT and COMMIT2, from the fiber density map. To fairly compare these different approaches, we normalized the connectomes and computed the distance between them and the normalized ground truth connectome. If we consider all connections, i.e., ε, then the error with COMMIT2 is very small as compared with the raw tractogram, LiFE, and COMMIT, which are about 50 to 80% higher, while SIFT and SIFT2 obtained even higher errors. Focusing only on the true-positive connections, i.e., ε_TP_, we observe again that COMMIT2 outperforms all other methods, but now SIFT2, LiFE, and COMMIT show comparable results, whereas the raw and SIFT-filtered ones have almost twice the error.

### Comparison to other basic filtering procedures

**Fig. 3:**
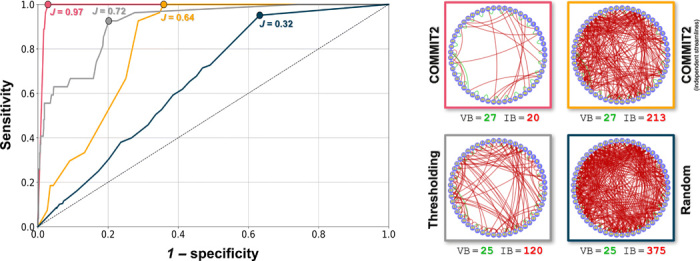
Comparison with basic filtering procedures. Receiver operating characteristic curve analysis to assess how COMMIT2 (pink) compares to other filtering procedures present in the literature in terms of discriminating between true-positive and false-positive bundles in the connectome. The yellow results correspond to using the same formulation of COMMIT2 but considering all streamlines as independent. We also tested the effect of filtering the tractogram by thresholding the bundles as function of their cardinality (gray) and their random removal using the same rate (dark blue). The best performance of each approach (i.e., max *J*) is reported as a graph for visual inspection.

We also compared COMMIT2 with other basic filtering procedures often used in the literature to discriminate between true-positive and false-positive bundles in the connectomes. Figure 3 reports the receiver operating characteristic curve analysis for the performance of these methods on the tractogram reconstructed with probabilistic tractography; results hold for deterministic tracking. The connectomes corresponding to the best performance (i.e., max *J*) of each method are reported as well. COMMIT2 results are plotted in pink as function of the relative importance of the anatomical priors in the filtering procedure (see Materials and Methods); the best performance with *J* = 0.97 corresponds to the connectome shown in Fig. 1D. The yellow line refers to results obtained by using the same formulation of COMMIT2 but considering the streamlines as independent (see Materials and Methods). This is called lasso regularization (*22*) and consists in promoting sparsity at the level of the individual streamlines rather than the bundles; this form of regularization was tested in the original COMMIT formulation (*12*). The gray line corresponds to filtering the tractogram by thresholding the bundles as function of their cardinality, i.e., removing progressively the connections containing a low number of streamlines, which is a very common procedure used in clinical studies to reduce the presence of false positives in the connectomes. For the sake of comparison, we also tested the effect of randomly filtering the bundles at the same removal rate of COMMIT2 (dark blue); the reported values correspond to the average score from 100 different experiments. The best performance of each approach (i.e., max *J*) is also reported as a graph for visual inspection. Comparing the pink and the yellow curves, we can easily appreciate that without the grouping of streamlines implemented in our new formulation, only a very modest improvement of the sensitivity/specificity trade-off was possible (max *J* = 0.64). COMMIT2 outperformed thresholding, as this latter could only improve marginally the initial configuration (VB = 27 and IB = 441; Fig. 2A, top left) up to a maximum *J* = 0.72, corresponding not only to decreasing the IB to 120 but also to a loss of two valid ones. Per contra, COMMIT2 was able to reduce considerably more IBs (441 → 20) before losing valid ones. We also investigated the impact of thresholding on the connectomes already filtered by previous methods (fig. S2). All methods largely benefitted from this additional postprocessing, even though none could reach the same performance of COMMIT2. Notably, thresholding caused the loss of 2 of the 27 VBs in all cases but not COMMIT2, on which it had no effect.

### Qualitative evaluation on in vivo human brain data

**Fig. 4:**
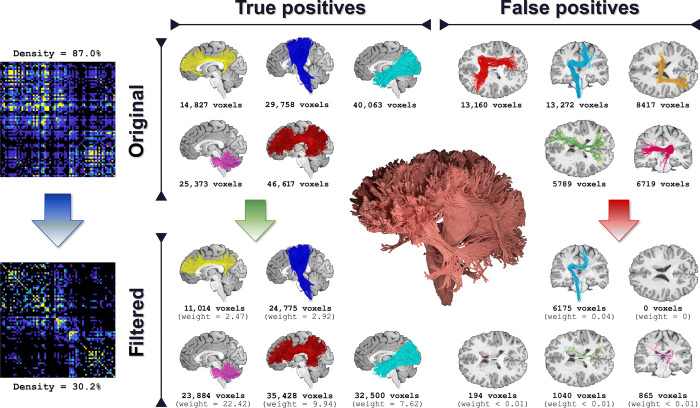
Qualitative evaluation of the proposed method with in vivo data. Top row: Connectome and bundles of the input tractogram; bottom row: the same filtered by COMMIT2. To better compare the bundles before and after the filtering, we report the voxel coverage and the sum of the streamline weights assigned by COMMIT2. The filtering procedure behaves extremely differently for the true (first column) and the false (second column) positives. While the voxel coverage of the true positives remains intact, the one of the false positives is dramatically reduced, and in some cases, they are completely removed. Looking at a more quantitatively meaningful measure—the COMMIT2 weights—we also appreciate that the weights associated with the true positives are much higher than those of the false positives, which are very close to zero.

Last, we tested the effectiveness of the new formulation on in vivo data from the Human Connectome Project (HCP) (*23*). As the ground truth is unknown, we qualitatively assessed the impact of COMMIT2 on known true-positive and false-positive bundles that were manually defined by an expert neuroanatomist. The top row of Fig. 4 shows these known bundles as reconstructed in the original tractogram along with the voxel coverage, i.e., number of voxels traversed by the streamlines associated with each bundle; the bottom row reports the same bundles after filtering with COMMIT2. Results show that our COMMIT2 filtering procedure does not affect the true-positive bundles, as the voxel coverage is comparable with the original tractogram and the total contributions estimated by COMMIT2 are high. This means that COMMIT2 removes implausible streamlines inside the bundles but recognizes these bundles as fundamental to explain the data and keeps the coverage of the white matter intact. Conversely, the false-positive bundles are markedly thinned, i.e., the voxel coverage is extremely reduced, and the weights assigned to them by COMMIT2 are close to zero, meaning they are not necessary to explain the signal. In particular, we observe that while the less populated bundles are (almost) completely removed, the more populated ones are extremely thinned in terms of white matter coverage and that their contribution to the resulting connectome is minimal. This effect can be observed also on the differences between the weights of the connectomes reported on the left side of the figure.

In the absence of a ground truth, we could only evaluate the estimated networks qualitatively. The raw connectome appears very dense, which is an expected result as it corresponds to probabilistic tracking, but we can clearly observe that after filtering with COMMIT2, it is definitely more sparse, in agreement with the recent theory about the economy of brain networks (*20*). The connectomes recovered with SIFT, SIFT2, LiFE, and COMMIT are shown in fig. S3; all have a density that is at least twice as dense than the one estimated with COMMIT2. However, from the visual inspection of the known true-positive and false-positive bundles, our results suggest that this higher level of sparsity does not imply the loss of valid connections but seem to indicate that the connections that are filtered out are indeed incompatible with the underlying data.

## DISCUSSION

Over the years, tractography has proven particularly effective for noninvasively studying the neuronal architecture of the brain, but recent studies have challenged its accuracy (*7*–*10*). In particular, it was shown that the presence of false-positive connections in the reconstructions can significantly bias the topological properties of the estimated brain networks, raising serious concerns for its use in mapping the human connectome. Some authors prescribed the need for a revolution of tractography techniques to reliably reconstruct the known anatomy while controlling for false positives and, particularly, that the notion of both anatomy and microstructure is essential to progress (*8*). We explicitly developed COMMIT2 with this concept in mind, and in fact, our novel formulation naturally incorporates both these characteristics. Apart from sharing the same convex optimization procedure with the original COMMIT, the method presented here is an important improvement of the previous framework and not only an incremental variant. The possibility to inject priors about brain anatomy and its organization, and not only about microstructural properties, represents a powerful and novel way to tackle the false-positive problem in tractography and brain structural connectivity. When we compared the performance of COMMIT2 with other state-of-the-art filtering techniques (LiFE, SIFT, SIFT2, and COMMIT) (*12*–*15*), which are all similar in spirit to our proposed method but are purely data driven, none of them proved effective in reducing IBs. This clearly indicates that adding anatomical priors about the organization of the bundles has indeed a dramatic impact on the specificity of the estimated connectomes.

COMMIT2 is not a pure tractography algorithm but a flexible filtering procedure that can be applied on top of any tractogram. An appealing feature of the COMMIT2 formulation is that it allows injecting additional priors on the bundles (see Materials and Methods). A possible way to take advantage of this property is to use the anatomical knowledge we have on bundles. In practice, if we knew that a connection surely exists, e.g., from atlases or population studies, then we could promote the corresponding bundle; conversely, if we knew that a specific tractography algorithm is keen to find a particular implausible connection, then we may want to penalize it more than others during the filtering. Thus, as our knowledge about true-positive and false-positive bundles improves, COMMIT2 results can be directly improved, since the framework can selectively promote or penalize groups of fibers with these priors. Besides, we demonstrated the effectiveness of this new formulation by fitting the streamlines to the intra-axonal signal fraction map estimated from DW-MRI with a specific biophysical model, but multiple options are available in the literature, e.g., (*24*–*26*), among others. We believe this choice does not affect the validity of our results, as this map could be replaced by any additive quantity as long as this provides a proxy measure of axonal density or any microstructural property of the fibers that is invariant along their pathway. The investigation of which map is the most suitable as input for COMMIT2 was beyond the scope of this work and will be the subject of future studies. Clearly, the more our knowledge on microstructural modeling grows, the more accurate the estimates with our framework will become.

By performing experiments with both deterministic and probabilistic tractography, we could observe that despite the initial connectomes were quite different, after filtering them with COMMIT2, they became more comparable and with minimal discrepancy of network density. On the contrary, the connectomes estimated by other methods differ quite heavily depending on the input tracking method. This suggests that the application of COMMIT2 converges toward a more reliable estimation of connectivity. Sarwar *et al.* (*27*) recently investigated whether using deterministic or probabilistic tracking is preferable for clinical studies. They concluded that to minimize the impact of false positives, one should prefer deterministic tracking at the price of having maybe some false negatives, unless a strong thresholding is used on the connectomes estimated with probabilistic tracking. Here, we presented results using both, showing that although the input was different, the final results obtained with COMMIT2 are comparable. On the tractogram obtained with probabilistic tracking, we also compared the performances of COMMIT2 against a standard thresholding procedure, showing that although the latter was able to improve the initial configuration, its best performance was far from the accuracy obtained with COMMIT2. In the same spirit, to analyze in vivo data, we performed probabilistic tracking with multishell multitissue anatomically constrained tractography (*28*) to highlight the benefits of the application of COMMIT2 even on top of one of the most accurate state-of-the-art methods. Nevertheless, we stress that, as already pointed out by the results on synthetic data, the performances of COMMIT2 on a different input tractogram would be comparable.

Besides improving the estimation of brain connectivity, we believe that COMMIT2 is also a promising tool that can be routinely used by neurosurgeons for whom tractography has rapidly become an essential tool for planning surgery. By allowing visualization of the different bundles before the operation, tractography has always been considered as a potential tool, but since its reliability has always been under debate, not all neurosurgeons feel comfortable to trust it. Nevertheless, the continuous progress in MRI scanners has already provided them an easy access to patient’s diffusion images through which they can now have access to a more reliable structural connectivity reconstruction. The short computational time required by COMMIT2 can allow its application on any type of whole-brain tractograms that may be built from a large database (like HCP www.humanconnectome.org, UK BioBank www.ukbiobank.ac.uk, …) to extract and study the most reliable bundles and omit the false-positive ones. With COMMIT2, we could now perform studies on the variability of the diffusion metrics along bundles in large cohorts of subjects and possibly relate them with their functional counterpart.

Although we obtained outstanding results, we acknowledge that the proposed framework is not without limitations, and there is room for future improvements. First, the bundle regularization guarantees that if a group is not necessary to explain the signal, then all its streamlines will be discarded. This implies that none of the eventual good streamlines present in that group will be kept, so the choice made to group streamlines plays a critical role. Instead, if a group is necessary to explain the signal, even if a streamline follows a very different path from the others in the same group because of this choice of regularization, then it will be kept since it still connects the same two regions of interest (ROIs). Although a proper way to filter inside groups is still under investigation and will be an object of future works, we can speculate that one possible way to do that is considering a finer parcellation for the gray matter, resulting in smaller groups to be evaluated by the framework. Another way could be using clustering techniques to group streamlines together, e.g., (*29*). All these possibilities will be tested and compared in future analyses. Another key assumption of our formulation is that, at the current resolution of DW-MRI and recalling that streamlines do not represent single axons, the contribution of every streamline remains constant along its path. This assumption is shared by all methods considered in this study and most state-of-the-art tractography algorithms present in the literature. Of course, if this assumption was not biologically valid, all these algorithms and the results presented here might be biased.

### Conclusions

In conclusion, our novel processing framework has the potential of changing the landscape of connectome analysis and, most importantly, improves our confidence in the interpretation of group differences or disease differences of certain connections in the connectomes, which now are characterized with more informative anatomical and quantitative microstructural properties. Tractography is not doomed and not inherently limited to choose a trade-off between sensitivity and specificity. COMMIT2 can break this trade-off by including a notion of local density of fibers and a group notion of bundles. This is more than an incremental improvement to tractography. It is a powerful step forward, which greatly improves the specificity of tractography algorithms and opens the door to quantitative and accurate analyses of the human connectome.

## MATERIALS AND METHODS

### Microstructure informed tractography

Given a DW-MR image I and a tractogram T, the acquired data can be seen as I=A(T)+η, where A;T→I is an operator describing the signal contribution of each fiber to the *n_d_* q-space samples acquired in the *n_v_* = *n_x_n_y_n_z_* voxels of $I\in {\mathbb{R}}_{+}^{n_{x}\times n_{y}\times n_{z}\times n_{d}}$ and η is the acquisition noise. The goal of tractography is to solve the inverse problem, i.e., finding the set of streamlines $\tilde{T}$ that best describes the acquired image I. The term “microstructure informed tractography” refers to a relatively novel area of research (*30*) whose aim is to obtain more quantitative and biologically meaningful estimates of brain connectivity by complementing tractography with biophysical models of the tissue microstructure (*31*). Several solutions have been proposed (*12*–*15*), but the originality of COMMIT (*12*) lies in the possibility to express tractography and tissue microstructure in a unified framework and solve this inverse problem using convex optimization. The signal in each voxel of I is described as a linear combination of the diffusion arising from all the fibers of T that intersect the voxel, in addition to local contributions from other tissues, e.g., cerebrospinal fluid (CSF). The joint problem can then be expressed as a system of linear equations

y=Ax+η (1)

where the vector $y\in {\mathbb{R}}_{+}^{n_{d}n_{v}}$ contains the *n_d_* DW-MR measurements acquired in the *n_v_* voxels of I, the matrix $A\;\in {\mathbb{R}}^{n_{d}n_{v}\times n_{c}}$ encodes the potential contributions of all streamlines in T (and possibly other tissues) to the signal in each voxel according to a given multicompartment model, and η accounts for both acquisition noise and modeling errors. The positive weights $x\in {\mathbb{R}}_{+}^{n_{c}}$ represent the actual contributions of the *n_c_* compartments, encoded as columns of A, needed to explain the acquired data I and can be estimated using non-negative least squares (NNLS)

$$\underset{x\geq 0}{\text{argmin}}\;{\left\|Ax-y\right\|}_{2}^{2}$$ (2)

where ||·||_2_ is the Euclidean norm in ℝ*^n^*.

Any multicompartment model (*31*–*33*) can be virtually used in COMMIT. In general, a multicompartment model assumes different diffusion behaviors according to the geometrical microstructure properties. For brain tissues, a common assumption is to distinguish between two or three compartments: intra-axonal (IA; mimicking the restricted movement of water molecules inside axons), extra-axonal (EA; mimicking the hindered movement outside axons), and isotropic (ISO; mimicking the free movement of the water like in CSF) if three are considered. The linear operator A is typically a block matrix of this form

$$A=[A^{\text{IA}}|A^{\text{EA}}|A^{\text{ISO}}]$$ (3)

where *n_c_* = *n_r_* + *n_h_* + *n_i_* and the submatrices $A^{\text{IA}}\in {\mathbb{R}}^{n_{d}n_{v}\times n_{r}}$, $A^{\text{EA}}\in {\mathbb{R}}^{n_{d}n_{v}\times n_{h}}$, and $A^{\text{ISO}}\in {\mathbb{R}}^{n_{d}n_{v}\times n_{i}}$ encode, respectively, the *n_r_* restricted, *n_h_* hindered, and *n_i_* isotropic contributions to the image. This formulation assumes invariance of a microstructural parameter (e.g., intra-axonal signal fraction and axon diameter) along a particular pathway and uses this prior to get more robust estimates of both the trajectory and microstructural properties of a fiber.

### Illustrative toy example

**Fig. 5:**
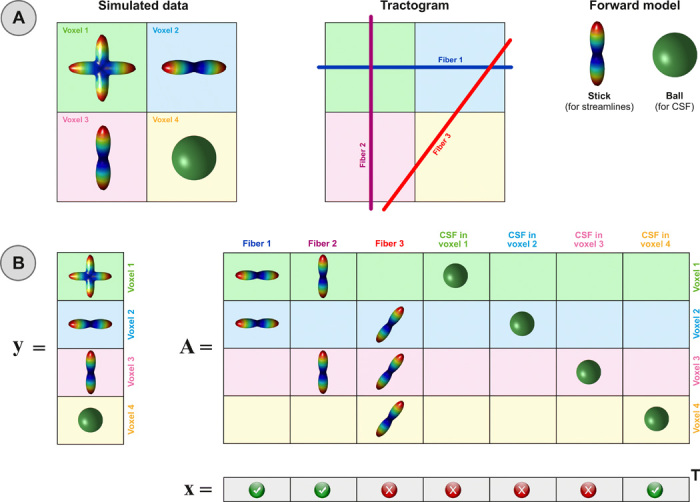
Synthetic toy example to illustrate the modeling and the parameter estimation using the COMMIT framework. (**A**) The simulated ODFs, a possible tractogram estimated with a generic tractography algorithm, and the forward model used to associate a signal contribution to each streamline. (**B**) The corresponding vector **y** containing the simulated data in all voxels, the matrix A encoding the signal contributions of each streamline according to the chosen forward model (and the potential presence of CSF), and the coefficients **x** estimated by COMMIT.

To illustrate this estimation process, let us consider the synthetic toy example shown in Fig. 5A. In the left panel, we display the orientation distribution functions (ODFs) simulated in each voxel, which were used to reconstruct the three streamlines visualized in the middle panel using a generic tractography algorithm. The right panel shows the forward model we adopted to construct the matrix A: a “stick” to account for the anisotropic contributions of the streamlines and a “ball” to consider possible CSF contaminations (*31*). Figure 5B illustrates the components of the linear system **y** = A**x** that we want to solve using COMMIT. In the column vector **y**, we concatenate the data simulated in each voxel. The matrix A is constructed by first checking how the reconstructed streamlines intersect the voxels: Fiber 1 crosses voxels 1 and 2, fiber 2 crosses voxels 1 and 3, and fiber 3 crosses voxels 2, 3, and 4. We then create one column for each streamline and store in the rows corresponding to each voxel it traverses the contribution of a stick oriented in the same direction of the streamline; if a streamline does not cross a voxel, then the corresponding rows are set to 0. To account for the possible presence of CSF in a voxel, we add four columns, and in each of them, we put 0 everywhere, except in the rows corresponding to a distinct voxel, where we insert an isotropic contribution according to the ball model.

Every column in A is controlled by a different contribution in **x**, and for a given configuration of contributions **x**, the predicted signal is obtained by performing the multiplication A**x**. COMMIT seeks for the optimal configuration of **x**, which must be positive, such that the predicted signal, i.e., A**x**, is as close as possible to the measured signal, i.e., **y**; hence, it tries to minimize their difference, i.e., $\text{argmin}\;{\left\|Ax-y\right\|}_{2}^{2}$. According to matrix-vector multiplication properties, we can immediately notice that to obtain the correct profile in voxel 1, we must have a positive contribution in the first two entries of **x** but 0 in x_4_, since there is no CSF in voxel 1. To assign the values to the remaining entries of **x**, we continue the multiplication. Looking at voxel 2, we observe that x_3_ = x_5_ = 0, while from the third and forth voxels, we obtain x_6_ = 0 and x_7_ = 1, respectively. The entries of **x** are uniquely determined, and since x_3_ = 0, fiber 3 is marked as a false positive and removed from the tractogram.

### Injecting priors about brain anatomy and its organization

**Fig. 6:**
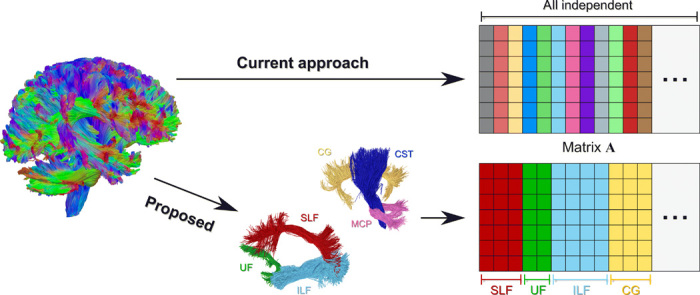
Injecting priors about brain anatomy and its organization. Current tractography algorithms consider all streamlines in a tractogram as independent entities, and COMMIT is no exception; every column of the matrix A encodes a different streamline, and all columns are treated as independent during the estimation of their contributions (**Top**). The proposed method (COMMIT2) groups streamlines belonging to the same anatomical bundle together and considers the corresponding columns of A as a single entity in the estimation; every streamline is still modeled by a distinct column, but streamlines belonging to the same bundle are arranged together as a sub-block of the matrix A and considered as a whole (**Bottom**). SLF, superior longitudinal fasciculus; UF, uncinate fasciculus; ILF, inferior longitudinal fasciculus; CG, cingulum; CST, cortico-spinal tract; MCP, middle cerebellar peduncle.

The purpose of this study was to evaluate whether we could improve the sensitivity/specificity trade-off of tractography by taking into consideration two fundamental observations about brain anatomy during the estimation process: (i) Streamlines are not “just lines” but represent neuronal fibers, and (ii) these neuronal fibers are naturally organized in bundles. To enforce the first prior knowledge, we implemented in A a simple forward model that assigns a contribution, i.e., volume or cross-sectional area, to each streamline of the input tractogram T proportionally to its length inside each voxel. Then, with Eq. 2, we require that the total amount of streamlines that traverse a voxel must sum up to the actual intra-axonal signal fraction in that voxel, which can be estimated in every voxel of the brain from DW-MR acquisitions using standard models like neurite orientation dispersion and density imaging (NODDI) (*26*) or spherical mean technique (SMT) (*25*). As each streamline represents a coherent set of real anatomical fibers, there cannot be a space for every possible reconstructed streamline. To implement the second prior, we first grouped all streamlines connecting the same pairs of gray matter regions and rearranged the corresponding columns of A accordingly, as shown in Fig. 6. Then, we added a new term to the cost function in Eq. 2 to try to explain the data, if possible, using the smallest number of these groups. Mathematically, this is achieved with the group lasso regularization (*34*), and the problem 2 becomes

$$\underset{x\geq 0}{\text{argmin}}\;{\left\|Ax-y\right\|}_{2}^{2}+\lambda \;\sum_{g\in G}{\left\|x^{\left(g\right)}\right\|}_{2}$$ (4)

where G is a general partition of the streamlines into groups, **x**^(*g*)^ represent the coefficients corresponding to the streamlines in a given group *g* ∈ G, and the parameter λ > 0 controls the trade-off between data and the regularization term. This additional term in the cost function penalizes the contributions at the level of groups and, in practice, promotes (but does not constrain) convergence toward a solution that explains the measured DW-MRI data with the minimum number of bundles; this formulation does not have any prior knowledge about which groups correspond to true-positive or false-positive bundles. Note that setting λ = 0 corresponds to the classical COMMIT. As this formulation represents an extension to the COMMIT framework, we refer to it as COMMIT2 in the manuscript.

Without any strong a priori knowledge on the bundles, a classical way to operatively solve this problem is to use the so-called adaptive group lasso (*35*), which penalizes all groups in the same manner regardless of their cardinality. The problem can then be rewritten as

$$\underset{x\geq 0}{\text{argmin}}\;{\left\|Ax-y\right\|}_{2}^{2}+\sum_{g\in G}\lambda^{\left(g\right)}\;{\left\|x^{\left(g\right)}\right\|}_{2}$$ (5)

with

$$\lambda^{\left(g\right)}=\frac{\lambda \;\sqrt{|g|}}{{\left\|x_{\;\text{NNLS}}^{\left(g\right)}\right\|}_{2}}$$ (6)

where ∣*g*∣ is the cardinality of the group *g* and $x_{\text{NNLS}}^{\left(g\right)}$ are the weights of the streamlines obtained by solving the NNLS problem in Eq. 2, i.e., without any regularization term.

### Evaluation criteria

We assessed the sensitivity and specificity of the resulting connectome using the Tractometer metrics defined in (*21*). True positives are described in terms of the valid connection (VC) ratio, which is the proportion of streamlines in the tractogram that connects a correct pair of ROIs, as well as the corresponding number of VBs. Similar metrics can be computed for the false positives, i.e., invalid connections (ICs) and IBs. To summarize sensitivity and specificity in a single score, we computed the Youden’s index *J* = sensitivity + specificity − 1. Sensitivity is defined as the ratio between VB and the number of real-positive bundles (27 in this dataset), and specificity as 1 − IB/*N*, where *N* is the number of real negatives (594 in this dataset). *N* represents the number of ROI pairs that may potentially be connected (incorrectly) by tractography and was computed by reconstructing 10 million streamlines with the probabilistic algorithm, as it is more permissive.

We also compared the performance of our novel formulation to other state-of-the-art filtering techniques: LiFE (*13*), SIFT (*14*), SIFT2 (*15*), and COMMIT (*12*). For each method, we downloaded the software package developed by the original authors—i.e., https://github.com/brain-life/encode for LiFE, https://mrtrix.org for SIFT/SIFT2, and https://github.com/daducci/COMMIT for COMMIT—and run the code with default parameters.

Last, we investigated the accuracy in the estimation of the actual weights of the edges in the ground truth connectome. However, as each technique assumes a different definition of connection strength between two brain regions, to validate these weights, we first normalized each connectome to its maximum value and then computed the root of the sum of squared differences between them and the normalized ground truth connectome. This measure was computed both considering all the connections in the connectomes

$$\varepsilon =\sqrt{\sum_{i,j=1}^{53}{\left(C_{i,j}-{\tilde{C}}_{i,j}\right)}^{2}}$$ (7)

and only the true-positive ones

$$\varepsilon_{\text{TP}}=\sqrt{\sum_{(i,j)\in \text{TP}}{\left(C_{i,j}-{\tilde{C}}_{i,j}\right)}^{2}}$$ (8)

where *C*_*i*, *j*_ indicate the entries of the ground truth connectome, ${\tilde{C}}_{i,j}$ indicate the entries obtained from one of the compared methods, and TP is a set containing only the pairs (*i*, *j*) corresponding to the true-positive bundles.

### Synthetic phantom description and processing

We quantitatively evaluated our novel approach using a realistic digital phantom with known ground truth developed for the reconstruction challenge organized in 2013 at the International Symposium on Biomedical Imaging (ISBI) using Phantomas (https://github.com/ecaruyer/phantomas). This simulated dataset is shown in Fig. 1A and consists of 27 ground truth fiber bundles that were specifically designed to mimic real fiber configurations typically encountered in the brain (Fig. 1B). These include complex arrangements of bending, crossing, and branching fibers at various angles and with different curvatures; in addition, three spherical regions corresponding to fast diffusive compartments such as in brain ventricles were added. The corresponding DW-MR signal was generated using the composite hindered and restricted model of diffusion (*24*) along 64 directions with *b* = 3000 s/mm^2^ and adding Rician noise with a signal-to-noise ratio of 30. The intra-axonal signal fraction of this phantom was computed from the geometry of the ground truth streamlines.

Connectomes were constructed from the reconstructions obtained with both deterministic and probabilistic tractography using the 53 gray matter ROIs as network nodes. We used the MRtrix software (*36*) as it is a popular processing suite to analyze DW-MR data. First, we computed the fiber orientation distributions (FODs) in each voxel using constrained spherical deconvolution (*37*), with l_max_ = 8. Then, we reconstructed 1 million streamlines with both deterministic (SD_STREAM) and probabilistic (iFOD2) algorithms using default parameters and performed the tracking using the white matter mask as the seed region. Last, we assigned each end point of a streamline to a node if that point fell within 2 mm from one of the 53 gray matter ROIs (default setting). A streamline was considered as connecting two nodes if both end points were assigned; otherwise, it was discarded and excluded from the analysis.

For completeness, we also evaluated the proposed framework using two additional digital phantoms with more complex network configurations, created with Phantomas following a similar procedure as described in the recent work of Sarwar *et al.* (*27*). We defined 20 gray matter ROIs and then randomly generated two three-dimensional geometries of fiber bundles to obtain a connection density in the resulting connectomes of 10% (fig. S1, blue panel) and 20% (fig. S1, rose panel), respectively. The centerline of each fiber bundle was defined using third-order piecewise polynomials, and a constant radius was randomly assigned to each bundle in the range of 1.5 to 4 mm. The generation of the DW-MR images and the processing were performed as before.

### In vivo data processing

We also tested COMMIT2 on in vivo data using data from the HCP repository (www.humanconnectome.org). We downloaded the preprocessed diffusion data corresponding to subject 100307 and the structural T1-weighted image with the corresponding standard Desikan-Killiany (*38*) parcellation in 85 gray matter ROIs performed with FreeSurfer (http://surfer.nmr.mgh.harvard.edu/). Detailed processing methods applied to all HCP open-access data are described in (*23*). We performed whole-brain anatomically constrained tractography (*28*). To do so, we first segment the T1-weighted image using FMRIB’s automated segmentation tool (*39*) to derive the multitissue image. This allowed performing the tissue-informed spherical deconvolution (*40*). With the recovered fiber orientation distributions, we performed probabilistic tracking (iFOD2) and the white matter mask as the seed region. We generated 5 million streamlines of length between 20 and 200 mm and default parameters. To create the connectome, we then used the standard 85 ROIs of the FreeSurfer Desikan-Killiany atlas (*38*), replacing the brainstem with only its last part (i.e., medulla). Among all the existing models to estimate the voxel-wise intra-axonal signal fraction, we decided to use the SMT (*25*). We acknowledge that this choice is arbitrary, but we believe it does not affect the validity of our framework. To improve the microstructure model is out of the scope of COMMIT2, although it is important to choose one that has been proven to be valid for the DW-MRI data regime identified by the acquisition’s parameters. We performed the fitting with the open-source code available at https://github.com/ekaden/smt. The connectomes were constructed using the streamline count for the original tractogram and the sum of weights for the one filtered with COMMIT2; we also computed the network density, i.e., the ratio between the actual and the possible connections. Figure S3 compares the connectomes obtained after applying the state-of-the-art methods (SIFT, SIFT2, LiFE, and COMMIT) as described in the previous sections. The processing time was ≈7^′^ for SIFT, ≈4^′^ for SIFT2, ≈24 hours for LiFE, ≈26^′^ for COMMIT, and ≈37^′^ for COMMIT2; all experiments were conducted on an AMD 1950x workstation with 16 cores and 64-gigabyte RAM.

### Code and data availability

The numerical phantom used as validation is publicly available and can be downloaded from https://github.com/ecaruyer/phantomas. The in vivo MRI data used are those of subject 100307 of the HCP and are available at www.humanconnectome.org. The code is open source and freely available at https://github.com/daducci/COMMIT.

## Supplementary Material

aba8245_SM.pdf

## SUPPLEMENTARY MATERIALS

Supplementary material for this article is available at http://advances.sciencemag.org/cgi/content/full/6/31/eaba8245/DC1

## Appendix

View/request a protocol for this paper from *Bio-protocol*.

## References

[1] Basser P. J., Pajevic S., Pierpaoli C., Duda J., Aldroubi A., In vivo fiber tractography using DT-MRI data. Magn. Reson. Med. 44, 625–632 (2000). 11025519 10.1002/1522-2594(200010)44:4<625::aid-mrm17>3.0.co;2-o

[2] Sporns O., Tononi G., Kötter R., The human connectome: A structural description of the human brain. PLOS Comput. Biol. 1, e42 (2005). 16201007 10.1371/journal.pcbi.0010042 PMC1239902

[3] Bassett D. S., Sporns O., Network neuroscience. Nat. Neurosci. 20, 353–364 (2017). 28230844 10.1038/nn.4502 PMC5485642

[4] Bullmore E. T., Bassett D. S., Brain graphs: Graphical models of the human brain connectome. Annu. Rev. Clin. Psychol. 7, 113–140 (2011). 21128784 10.1146/annurev-clinpsy-040510-143934

[5] Bassett D. S., Bullmore E. T., Human brain networks in health and disea**s**e. Curr. Opin. Neurol. 22, 340–347 (2009). 19494774 10.1097/WCO.0b013e32832d93dd PMC2902726

[6] Griffa A., Baumann P. S., Thiran J.-P., Hagmann P., Structural connectomics in brain diseases. Neuroimage 80, 515–526 (2013). 23623973 10.1016/j.neuroimage.2013.04.056

[7] Thomas C., Ye F. Q., Irfanoglu M. O., Modi P., Saleem K. S., Leopold D. A., Pierpaoli C., Anatomical accuracy of brain connections derived from diffusion MRI tractography is inherently limited. Proc. Natl. Acad. Sci. U.S.A. 111, 16574–16579 (2014). 25368179 10.1073/pnas.1405672111 PMC4246325

[8] Maier-Hein K. H., Neher P. F., Houde J.-C., Côté M.-A., Garyfallidis E., Zhong J., Chamberland M., Yeh F.-C., Lin Y.-C., Ji Q., Reddick W. E., Glass J. O., Chen D. Q., Feng Y., Gao C., Wu Y., Ma J., He R., Li Q., Westin C.-F., Deslauriers-Gauthier S., González J. O. O., Paquette M., St-Jean S., Girard G., Rheault F., Sidhu J., Tax C. M. W., Guo F., Mesri H. Y., Dávid S., Froeling M., Heemskerk A. M., Leemans A., Boré A., Pinsard B., Bedetti C., Desrosiers M., Brambati S., Doyon J., Sarica A., Vasta R., Cerasa A., Quattrone A., Yeatman J., Khan A. R., Hodges W., Alexander S., Romascano D., Barakovic M., Auría A., Esteban O., Lemkaddem A., Thiran J.-P., Cetingul H. E., Odry B. L., Mailhe B., Nadar M. S., Pizzagalli F., Prasad G., Villalon-Reina J. E., Galvis J., Thompson P. M., De Santiago Requejo F., Laguna P. L., Lacerda L. M., Barrett R., Dell’Acqua F., Catani M., Petit L., Caruyer E., Daducci A., Dyrby T. B., Holland-Letz T., Hilgetag C. C., Stieltjes B., Descoteaux M., The challenge of mapping the human connectome based on diffusion tractography. Nat. Commun. 8, 1349 (2017). 29116093 10.1038/s41467-017-01285-x PMC5677006

[9] Zalesky A., Fornito A., Cocchi L., Gollo L. L., van den Heuvel M. P., Breakspear M., Connectome sensitivity or specificity: Which is more important? Neuroimage 142, 407–420 (2016). 27364472 10.1016/j.neuroimage.2016.06.035

[10] Drakesmith M., Caeyenberghs K., Dutt A., Lewis G., David A. S., Jones D. K., Overcoming the effects of false positives and threshold bias in graph theoretical analyses of neuroimaging data. Neuroimage 118, 313–333 (2015). 25982515 10.1016/j.neuroimage.2015.05.011 PMC4558463

[11] Schilling K. G., Nath V., Hansen C., Parvathaneni P., Blaber J., Gao Y., Neher P., Aydogan D. B., Shi Y., Ocampo-Pineda M., Schiavi S., Daducci A., Girard G., Barakovic M., Rafael-Patino J., Romascano D., Rensonnet G., Pizzolato M., Bates A., Fischi E., Thiran J.-P., Canales-Rodríguez E. J., Huang C., Zhu H., Zhong L., Cabeen R., Toga A. W., Rheault F., Theaud G., Houde J.-C., Sidhu J., Chamberland M., Westin C. F., Dyrby T. B., Verma R., Rathi Y., Irfanoglu M. O., Thomas C., Pierpaoli C., Descoteaux M., Anderson A. W., Landman B. A., Limits to anatomical accuracy of diffusion tractography using modern approaches. Neuroimage 185, 1–11 (2019). 30317017 10.1016/j.neuroimage.2018.10.029 PMC6551229

[12] Daducci A., Dal Palù A., Lemkaddem A., Thiran J.-P., COMMIT: Convex optimization modeling for microstructure informed tractography. IEEE Trans. Med. Imaging 34, 246–257 (2015). 25167548 10.1109/TMI.2014.2352414

[13] Pestilli F., Yeatman J. D., Rokem A., Kay K. N., Wandell B. A., Evaluation and statistical inference for human connectomes. Nat. Methods 11, 1058–1063 (2014). 25194848 10.1038/nmeth.3098 PMC4180802

[14] Smith R. E., Tournier J. D., Calamante F., Connelly A., SIFT: Spherical-deconvolution informed filtering of tractograms. Neuroimage 67, 298–312 (2013). 23238430 10.1016/j.neuroimage.2012.11.049

[15] Smith R. E., Tournier J. D., Calamante F., Connelly A., SIFT2: Enabling dense quantitative assessment of brain white matter connectivity using streamlines tractography. Neuroimage 119, 338–351 (2015). 26163802 10.1016/j.neuroimage.2015.06.092

[16] Smith R. E., Tournier J.-D., Calamante F., Connelly A., The effects of SIFT on the reproducibility and biological accuracy of the structural connectome. Neuroimage 104, 253–265 (2015). 25312774 10.1016/j.neuroimage.2014.10.004

[17] Jones D. K., Knösche T. R., Turner R., White matter integrity, fiber count, and other fallacies: The do’s and don’ts of diffusion MRI. Neuroimage 73, 239–254 (2013). 22846632 10.1016/j.neuroimage.2012.06.081

[18] Delettre C., Messé A., Dell L.-A., Foubet O., Heuer K., Larrat B., Meriaux S., Mangin J.-F., Reillo I., de Juan Romero C., Borrell V., Toro R., Hilgetag C. C., Comparison between diffusion MRI tractography and histological tract-tracing of cortico-cortical structural connectivity in the ferret brain. Netw. Neurosci. 3, 1038–1050 (2019). 31637337 10.1162/netn_a_00098 PMC6777980

[19] van den Heuvel M. P., de Reus M. A., Barrett L. F., Scholtens L. H., Coopmans F. M. T., Schmidt R., Preuss T. M., Rilling J. K., Li L., Comparison of diffusion tractography and tract-tracing measures of connectivity strength in rhesus macaque connectome. Hum. Brain Mapp. 36, 3064–3075 (2015). 26058702 10.1002/hbm.22828 PMC6869766

[20] Bullmore E., Sporns O., The economy of brain network organization. Nat. Rev. Neurosci. 13, 336–349 (2012). 22498897 10.1038/nrn3214

[21] Côté M.-A., Girard G., Boré A., Garyfallidis E., Houde J.-C., Descoteaux M., Tractometer: Towards validation of tractography pipelines. Med. Image Anal. 17, 844–857 (2013). 23706753 10.1016/j.media.2013.03.009

[22] Tibshirani R., Regression shrinkage and selection via the lasso. J. R. Stat. Soc. B. Methodol. 58, 267–288 (1996).

[23] Van Essen D. C., Smith S. M., Barch D. M., Behrens T. E. J., Yacoub E., Ugurbil K.; WU-Minn HCP Consortium, The WU-Minn human connectome project: An overview. Neuroimage 80, 62–79 (2013). 23684880 10.1016/j.neuroimage.2013.05.041 PMC3724347

[24] Assaf Y., Basser P. J., Composite hindered and restricted model of diffusion (CHARMED) MR imaging of the human brain. Neuroimage 27, 48–58 (2005). 15979342 10.1016/j.neuroimage.2005.03.042

[25] Kaden E., Kelm N. D., Carson R. P., Does M. D., Alexander D. C., Multi-compartment microscopic diffusion imaging. Neuroimage 139, 346–359 (2016). 27282476 10.1016/j.neuroimage.2016.06.002 PMC5517363

[26] Zhang H., Schneider T., Wheeler-Kingshott C. A., Alexander D. C., NODDI: Practical in vivo neurite orientation dispersion and density imaging of the human brain. Neuroimage 61, 1000–1016 (2012). 22484410 10.1016/j.neuroimage.2012.03.072

[27] Sarwar T., Ramamohanarao K., Zalesky A., Mapping connectomes with diffusion MRI: Deterministic or probabilistic tractography? Magn. Reson. Med. 81, 1368–1384 (2019). 30303550 10.1002/mrm.27471

[28] Smith R. E., Tournier J.-D., Calamante F., Connelly A., Anatomically-constrained tractography: Improved diffusion MRI streamlines tractography through effective use of anatomical information. Neuroimage 62, 1924–1938 (2012). 22705374 10.1016/j.neuroimage.2012.06.005

[29] O’Donnell L. J., Golby A. J., Westin C.-F., Fiber clustering versus the parcellation-based connectome. Neuroimage 80, 283–289 (2013). 23631987 10.1016/j.neuroimage.2013.04.066 PMC3731058

[30] Daducci A., Dal Palú A., Descoteaux M., Thiran J.-P., Microstructure informed tractography: Pitfalls and open challenges. Front. Neurosci. 10, 247 (2016). 27375412 10.3389/fnins.2016.00247 PMC4893481

[31] Panagiotaki E., Schneider T., Siow B., Hall M. G., Lythgoe M. F., Alexander D. C., Compartment models of the diffusion MR signal in brain white matter: A taxonomy and comparison. Neuroimage 59, 2241–2254 (2012). 22001791 10.1016/j.neuroimage.2011.09.081

[32] Alexander D. C., Dyrby T. B., Nilsson M., Zhang H., Imaging brain microstructure with diffusion MRI: Practicality and applications. NMR Biomed. 32, e3841 (2019). 29193413 10.1002/nbm.3841

[33] Novikov D. S., Fieremans E., Jespersen S. N., Kiselev V. G., Quantifying brain microstructure with diffusion MRI: Theory and parameter estimation. NMR Biomed. 32, e3998 (2019). 30321478 10.1002/nbm.3998 PMC6481929

[34] Yuan M., Lin Y., Model selection and estimation in regression with grouped variables. J. R. Stat. Soc. Series B Stat. Methodology 68, 49–67 (2006).

[35] Wang H., Leng C., A note on adaptive group lasso. Comput. Stat. Data Anal. 52, 5277–5286 (2008).

[36] Tournier J.-D., Smith R., Raffelt D., Tabbara R., Dhollander T., Pietsch M., Christiaens D., Jeurissen B., Yeh C.-H., Connelly A., *Mrtrix3*: A fast, flexible and open software framework for medical image processing and visualisation. Neuroimage 202, 116137 (2019). 31473352 10.1016/j.neuroimage.2019.116137

[37] Tournier J.-D., Calamante F., Connelly A., Robust determination of the fibre orientation distribution in diffusion MRI: Non-negativity constrained super-resolved spherical deconvolution. Neuroimage 35, 1459–1472 (2007). 17379540 10.1016/j.neuroimage.2007.02.016

[38] Desikan R. S., Ségonne F., Fischl B., Quinn B. T., Dickerson B. C., Blacker D., Buckner R. L., Dale A. M., Maguire R. P., Hyman B. T., Albert M. S., Killiany R. J., An automated labeling system for subdividing the human cerebral cortex on MRI scans into gyral based regions of interest. Neuroimage 31, 968–980 (2006). 16530430 10.1016/j.neuroimage.2006.01.021

[39] Zhang Y., Brady M., Smith S., Segmentation of brain MR images through a hidden Markov random field model and the expectation-maximization algorithm. IEEE Trans. Med. Imaging 20, 45–57 (2001). 11293691 10.1109/42.906424

[40] Jeurissen B., Tournier J.-D., Dhollander T., Connelly A., Sijbers J., Multi-tissue constrained spherical deconvolution for improved analysis of multi-shell diffusion MRI data. Neuroimage 103, 411–426 (2014). 25109526 10.1016/j.neuroimage.2014.07.061

