# Supplementary material for: A new method for accurate in vivo mapping of human brain connections using microstructural and anatomical information
Source: Sci Adv. 2020 Jul 29;6(31):eaba8245. doi: 10.1126/sciadv.aba8245 (PMC7399649; doi:10.1126/sciadv.aba8245)
Supplement: aba8245_SM.pdf [file aba8245_SM.pdf]

Science Advances
AAAS

advances.sciencemag.org/cgi/content/full/6/31/eaba8245/DC1

Supplementary Materials for

A new method for accurate in vivo mapping of human brain connections using microstructural and anatomical information

Simona Schiavi, Mario Ocampo-Pineda, Muhamed Barakovic, Laurent Petit,
Maxime Descoteaux, Jean-Philippe Thiran, Alessandro Daducci*

*Corresponding author. Email: alessandro.daducci@univr.it

Published 29 July 2020, *Sci. Adv.* **6**, eaba8245 (2020)
DOI: 10.1126/sciadv.aba8245

This PDF file includes:

Figs. S1 to S4
Text S1

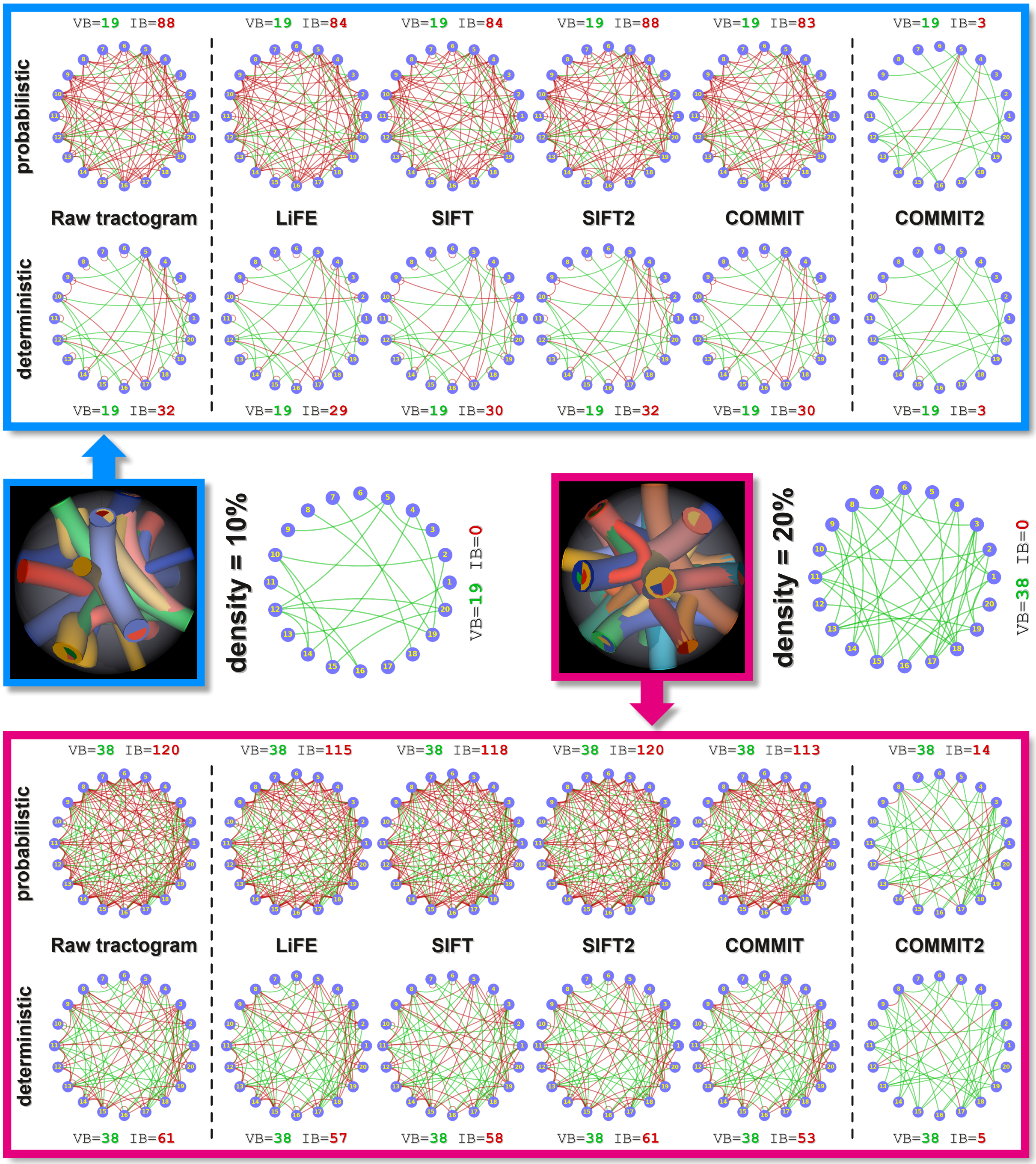

Fig. S1. Sensitivity and specificity of the connectomes in two additional digital phantoms with more complex and realistic network configurations. Similarly to Fig. 2A, we compare the valid (VB, green) and invalid (IB, red) bundles before and after applying all state-of-the-art filtering methods. The *blue* and the *rose* blocks correspond to the phantom with 10% and 20% connection density, respectively. COMMIT2 performances are systematically better than any other method in both configurations and show similar trends for both tractography algorithms.

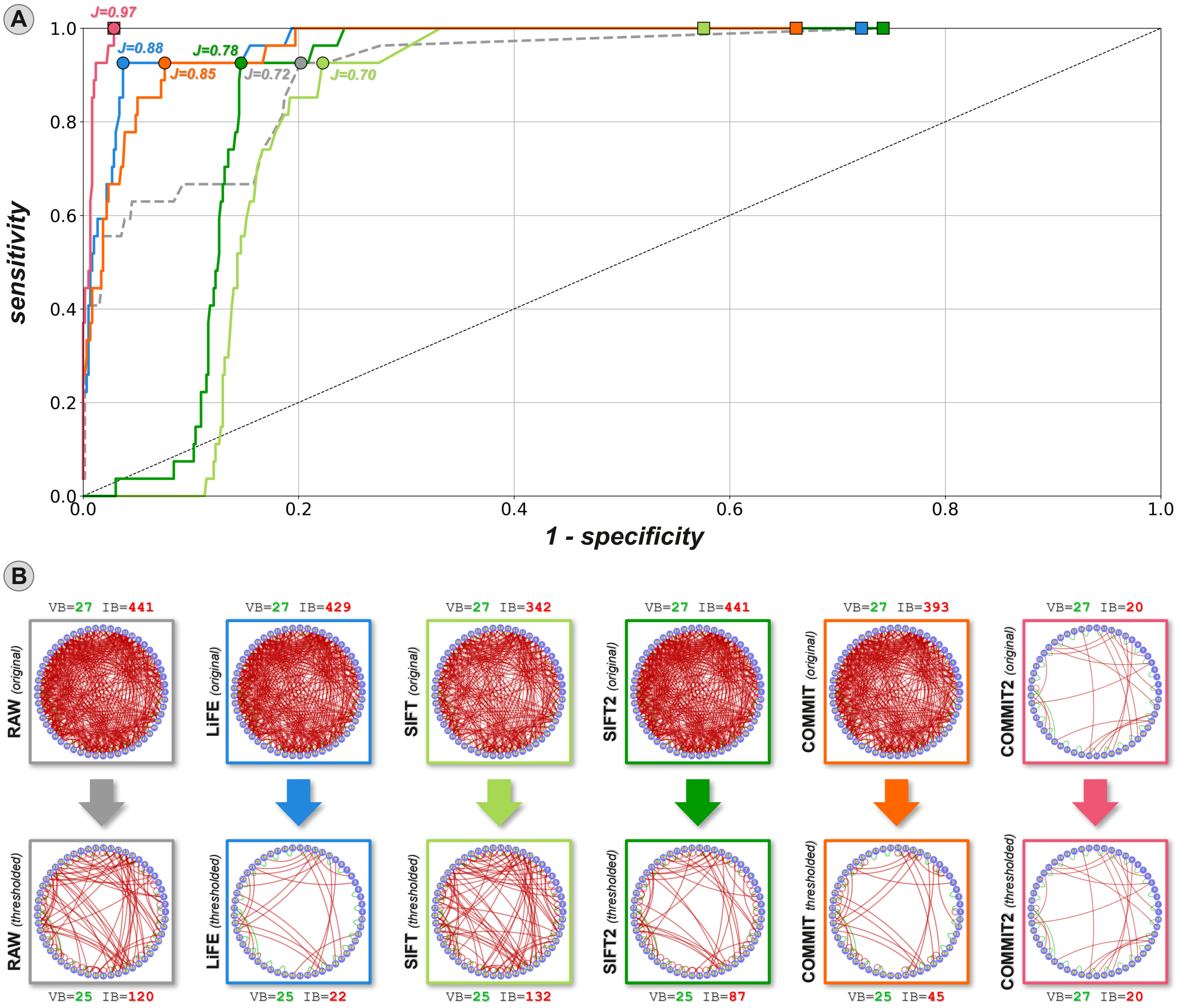

Fig. S2. Effect of thresholding on the estimated connectomes. (**A**) Receiver Operating Characteristic curve analysis to assess how COMMIT2 compares to the state-of-the-art filtering procedures when their estimated connectomes are further thresholded. Connections were gradually removed from the connectomes based on the streamline count for raw tractograms and SIFT, and on the streamline weight sum for LiFE, SIFT2, COMMIT and COMMIT2. The *squares* correspond to the original connectomes estimated by each method, whereas the *circles* to the best performance after thresholding, i.e. max J. The connectomes before and after this additional filtering are reported in panel (**B**) for visual inspection. All methods largely benefitted from this additional thresholding, even though none could reach the same performance of COMMIT2. Besides, thresholding caused the loss of 2 valid bundles for each method whereas it had no effect in case of COMMIT2.

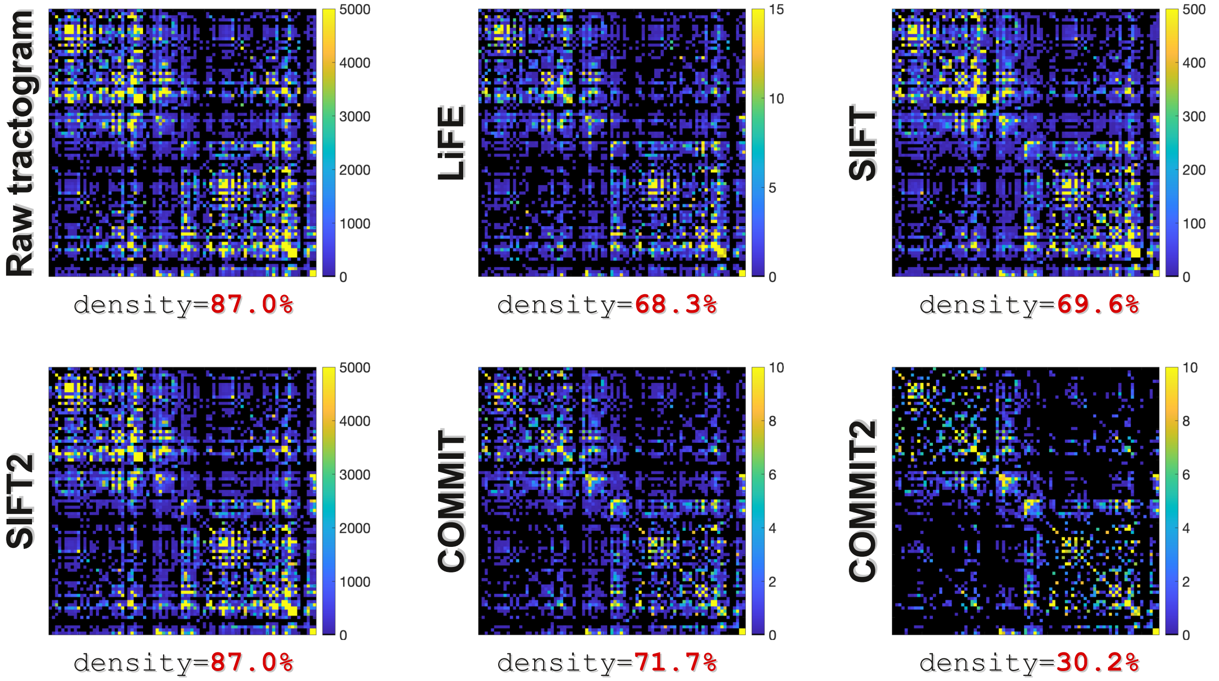

Fig. S3. Comparison of connectomes estimated on the HCP data. From the top left to the bottom right we show the connectomes estimated with probabilistic tractography before (first entry) and after applying state-of-the-art filtaering methods: LiFE (second entry), SIFT (third entry), SIFT2 (fourth entry) and COMMIT (fifth entry). Results with our novel filtering approach (COMMIT2) are reported in the last entry. Below each connectome the density of the network is reported.

Text S1

Optimization of the regularization parameter. To set the regularization parameter λ that scales the groups' penalization and gives the best performance in the synthetic phantom, we performed several experiments by starting from zero and iteratively incrementing its value. We thus analyze the behavior of the VB, IB, and Youden's index J. Fig. S4 analyzes the quality of the reconstructions that can be obtained by processing the raw tractogram using the proposed method. These plots correspond to probabilistic tracking, but similar results are obtained with the deterministic algorithm. In the first row are reported the number of VB and IB as a function of the regularization strength λ. Dashed lines correspond to the raw/unprocessed tractogram: VB=27 (corresponding to a sensitivity of 100%) and IB=441 (specificity 25.8%). We can notice that as λ increases, the number of IB decreases rather quickly but, correspondingly, the VB exhibit a much slower decrease trend. The decreasing rate of the IB slows down when they reach a value comparable with the VB. However, as expected, by increasing the regularization even further the number of VB also begins to decrease because, as it is known in optimization theory, when λ is too large the second term of Eq. 4 dominates and all groups are progressively discarded. To help to choose the optimal value for λ, we made use of the index J, which is shown in the second row along with the percentage of valid connections (VC). The maximum value of J is about 0.97, which corresponds to VB=27 (sensitivity 100%) and IB=20 (specificity 96.6%). After it reaches its maximum, J starts decreasing as more and more VB are suppressed. Nonetheless, it is interesting to note that the percentage of VC exhibits an increasing trend also after this value, indicating that the rate of decrease of the IB if faster than the VB.

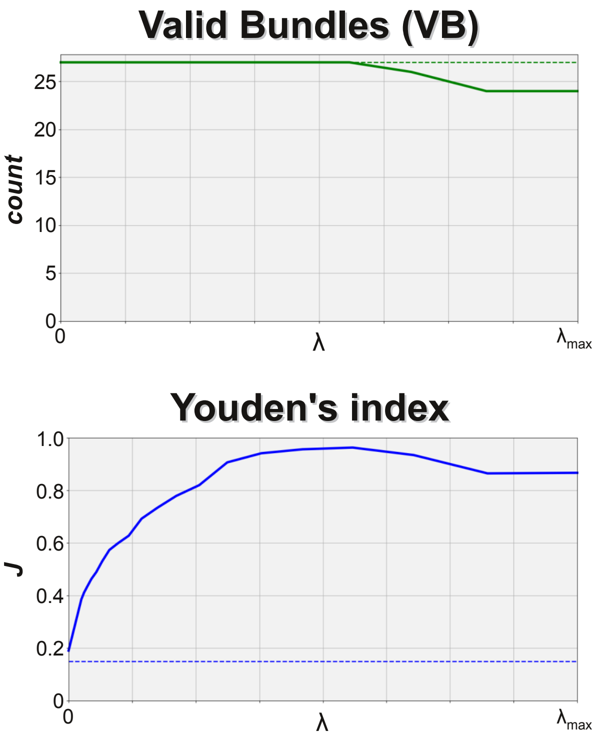

Fig. S4. Impact of adding the bundle-wise anatomical priors to COMMIT on the quality of the reconstructions. In the first row are reported the number of valid (VB) and invalid (IB) bundles as function of the regularization strength (λ). In the second row are reported the Youden's index and the percentage of valid connections (VC). Results correspond to probabilistic tractography.
